# Eclectic Review on Pulmonic Inflammation

**Published:** 1828-04-01

**Authors:** 


### Medico-Chirurgical Review,

### No. XVI.

[FASCICULUS I.]

JANUARY 12, 1828.

### Art. I.

### *Eclectic Review of the Etiology, Pathology, Diagnosis, and Treatment of Peripneumony.**

WHATEVER may be the case in the more genial climates of the earth, there can be no doubt that, under the gloomy skies of northern Europe, and especially of England, inflammation of the respiratory apparatus, acute and chronic, is at least three times more destructive of human life, than that of any other internal organ of the body. If we take into consideration the development of tubercles, and, consequently, of pulmonary phthisis, as materially influenced, if not produced, by peripneumonic inflammation, the ratio of its fatality will be greatly increased, and the importance of its study rendered more conspicuous. Common as is the disease in this country, we venture to affirm, that there are few internal inflammations in which more mistakes are made, as regards the seat, the extent, or the stage of the malady. Yet, on an accurate knowledge of these hinges the proper and successful treatment—and on the absence of this knowledge hangs the fate of the patient—sometimes the reputation of the practitioner.

If these observations be founded in truth, the utility of widely diffusing a rather extended article on this important subject will not be disputed. It is the first of a series of ECLECTIC REVIEWS on the more important diseases to which we are liable, and in which series we shall endeavour to concentrate the best information which literary research, modern pathology, and practical observation can yield.

It is well known that Cullen and Frank combined pleurisy and peripneumony, as either indistinguishable, or useless to be distinguished. Nothing can be more erroneous or improper than either of these conclusions. Dr. Good acknow-

* Laennec's Work—Dr. Forbes' Notes to Translation—Louis—Andral—Broussais, &c. &c.

ledged this; but his distinctive characters, contained in a few lines of the few pages dedicated to so important a disease, are totally inadequate, as founded on the plus or minus in certain symptoms, viz. the pulse, the respiration, the colour of the face, the cough, the expectoration, and the pain. Laennec goes on a much more accurate and scientific plan, investigating the disease through the medium of its anatomical characters, and its external physical signs, including auscultation and percussion. Him, therefore, we will chiefly follow in this article. The talented pathologist in question treats of peripneumony (by which he means exclusively inflammation of the pulmonary substance) under the following heads —acute peripneumony—partial peripneumony, and pulmonary abscess—gangrene of the lungs—chronic peripneumony—latent and symptomatic peripneumony.

### I.—ANATOMICAL CHARACTERS OF ACUTE PNEUMONIA.

These are divided by Laennec into three stages or degrees, distinctly marked, and easily recognized, viz. engorgement, hepatization, and infiltration.

1. ENGORGEMENT. "In this degree, the lung is externally of a livid or violet hue, heavier, and much more solid than natural. It is, however, still crepitous, but much less so than in a sound state, and, on pressing it between the fingers, we perceive that it is injected by a liquid. It retains the impression of the fingers nearly like an œdematous limb. When cut into, it appears of a livid or blood colour, is quite injected with a frothy serous fluid, more or less sanguineous, which flows from it abundantly. We can still, however, discover very clearly the natural alveolar and spongy texture of the viscus, except in some points, where the obstruction is more solid and compact, indicating the transition from the first to the second degree of peripneumony."

2. HEPATIZATION. In this stage, the lung has lost its crepitous feel, and resembles liver. It is often less livid externally than in engorgement, but exhibits internally a greater or less degree of redness, varying, in different parts, from violet-grey to blood-red, thus resembling some kinds of variegated marble, and contrasting strongly with the bronchia, the blood-vessels, the specks of black pulmonary matter, and the membranous partitions. If portions of lung in this state be cut into slices, hardly any fluid exudes; but if the incised surfaces are scraped with a scalpel, some bloody turbid serum is procured, intermixed with a whitish and puriform fluid. If inspected in a proper light, the pulmonary structure presents a granular aspect, instead of a cellular appearance. This is considered the characteristic anatomical feature of inflammation of the lung as discriminating it from tuberculation. The granular texture is still more observable when a portion of hepatized lung is torn. The granulation is produced by the conversion of the air-cells into solid bodies, with thickening of their parietes, and obliteration of their cavities by a concrete fluid. The hepatized lung seems, but is not really, larger than natural. The affected side does not measure more than the other—which is not the case in pleurisy.

3. INFILTRATION. "In this stage, the substance of the lung has the same degree of hardness, and the granular appearance above described, but is of a yellowish-pale, or straw colour. At first, the pus, as it begins to form, appears in small detached yellow points, increasing the motley-coloured shading formerly noticed. These points gradually combine, and the whole lung finally assumes a uniform straw, or lemon-yellow colour, and when incised exudes, in greater or less quantity, a yellow opaque, viscid matter, evidently purulent, but much less offensive to the smell than the pus of an external wound. In this state, the pulmonary substance is much more humid and softer than in the red hepatization. The granulated texture gradually disappears as the purulent softening advances : and even before this latter stage has attained its acmé, the parenchyma of the lungs gives way beneath the fingers like a soft clot. When the lung contains much black pulmonary matter, as is very commonly the case in adults and old persons, both the pus and the pulmonary substance assume an ash-grey colour, which has been recently denominated by some writers *grey hepatization.**"

The state above described is, strictly speaking, suppuration of the lung—the rare cases in which the pus is collected into a focus, constituting abscess, will be hereafter noticed.

The three stages or degrees are very commonly combined in various ways. Thus, one lung will be inflamed in the third degree throughout, while the other presents portions in the first or second stage. Nay, the three degrees are found in the same lung, partitioning it into zones, strongly contrasted, or insensibly blending into each other. Sometimes these indurations are precisely circumscribed by a pulmonary lobule; and in children especially, we occasionally find dispersed in the centre of the lungs, a certain number of lobules, arrived at the state of hepatization, while those immediately surrounding them are perfectly sound.

The lower portions of lungs are those most commonly occupied by peripneumony—a fact confirmed by Andral, who, in 88 cases of pneumonia, found 47 where the inferior lobe was affected—30 in which the upper was the seat of the disease—and 11 in which the whole lung was hepatized. Broussais, who is always opposed to Laennec, does not admit the proposition of the latter in its fullest extent—and Frank's experience is in the opposite direction. He found the upper more frequently affected than the inferior lobes—but Laennec is probably nearer the truth than any of them.

As might be expected, we never find the whole of both lungs inflamed in the third, or even in the second degree. Respiration could not be carried on till such a state obtained. But it is not uncommon to meet with one whole lung, and more than half the other, quite impervious to air. On the other hand, death often takes place before a fourth part of the respiratory apparatus becomes inflamed in any degree—shewing, as many other facts do, that death is frequently induced by exhaustion of the vital principle, rather than by the extent or intensity of the organic lesion. Morgagni, Baglivi, and many others, in former times, as well as Laennec, Andral, and recent pathologists, have observed that the right lung is much more frequently the seat of disease, of all kinds, than the left. This is hard to be accounted for, but it is a fact.

4. Abscess in the Lungs. Notwithstanding the opinions of ancients and moderns to the contrary, there is no organic lesion more uncommon than abscess, or what is termed vomica of the lungs. This vomica is, in reality, "the result of the softening of a large mass of tuberculous matter." In several hundred dissections of peripneumonia, made in a period of 20 years, M. Laennec has not met with more than five or six instances of "a collection of pus in an inflamed lung." These were not numerous, or of large extent in the same lung. They were dispersed in different parts of lungs arrived at the third stage (infil-

* "This term, which is incorrectly applied to the state described in the text, inasmuch as the colour is not *grey*, but *yellow-greyish*, or *ash-coloured*, is moreover improper, being applied to other morbid conditions of the lungs.—*Author.*"

tration) of inflammation. The parietes of these purulent depots were formed by the pulmonary tissue, infiltrated with pus, and in a state of soft disorganization, gradually decreasing as the centre was receded from.

"When we forcibly drag," says M. Laennec, "from the cavity of the chest an inflamed lung, attached to the costal pleura by old cellular adhesions, it frequently happens that the parts most infiltrated with pus give way under the fingers, or, without suffering any external wound, yield internally under the pressure, so as to form a soft sanious mass, which an inattentive observer might mistake for a collection of pus: if cases of this kind were received as instances of pulmonary abscess, nothing would be more common. Once only, during the space of time above-mentioned, have I seen a collection of pus in the lungs, of considerable extent. This was situated in the middle of the lung anteriorly, was flat and elongated, and would have contained three fingers. The walls of this abscess had, properly speaking, no surface, the pus being observed gradually to pass into a purulent detritus, and this into a firmer tissue, still loaded with pus, as we receded from the centre of the collection: and, at length, about half an inch from the matter, the purulent infiltration was not greater than it usually is in the third stage of the pulmonary inflammation. In this case, as in all the rest where abscess was found, the inflammation occupied only a part of one lung. This circumstance may help to account for the infrequency of collections of pus in the lungs, as cases of partial peripneumony usually yield either to nature or art, while an affection of great extent produces death, before the purulent infiltration is so far advanced as to form, by the destruction of the tissue containing it, distinct collections of pus."—*Forbes' Trans.*

The testimony of Broussais and Andral is very strong on this point. English pathologists, including Baillie himself, have fallen into numerous errors respecting pulmonary abscess, clearly from want of that minute attention to morbid alterations which characterizes the researches of the moderns, and especially of the Continental physicians.

There have been only two instances of pulmonary abscess presented to the public, during the last twenty years, by the French pathologists. In a preparation shewn by M. Honoré to the Royal Academy, there existed, in the centre of a hepatized lobe, an excavation filled with pus, and capable of containing a middle-sized apple. The patient had died of acute pneumonia. The other instance is given by M. Andral, and occurred in a man who died on the 19th day of pulmonic inflammation. The middle and lower lobes, on the right side, were in a state of purulent infiltration; and, towards the middle of the lower lobe, the tissue was found degenerated into a kind of paste, in the centre of which there was found pure pus. It is evident how much these differ from the softening down of tubercles.

"In these last, although the colour and appearance of the tuberculous matter are, in some cases, pretty much like those of pus, they generally differ, in containing tuberculous fragments, of a friable consistence. Besides the exact circumscription of the tuberculous excavations, the solidity of their walls, the soft false membrane with which these are constantly lined, and the semi-cartilaginous membrane which occasionally succeeds to this, suffice to discriminate these from the purulent collections above described; independently of the difference of the stethoscopic signs, which characterize them respectively in the living body."

Notwithstanding this rarity of pulmonary abscess, M. Laennec has met with two or three cases of enormous excavations, occupying nearly the whole of one lung, not apparently originating in softened tubercles.

RESOLUTION OF PERIPNEUMONY. Let it be remembered that our talented pathologist is still speaking in the language of morbid anatomy. When resolution takes place before the inflammation has reached the second stage, the effused blood is absorbed, and the pulmonary structure appears sound, but red, as if dyed. If the inflammation had reached the second stage, (hepatization) the indurated parts become of a pale reddish colour, the natural appearance of the lungs. But while this salutary change is taking place, the texture of the part becomes softer, more humid, and, when cut into, exudes more serum than blood —the serum being at first intermixed with small air-bubbles, gradually becoming more frothy. The granular aspect of the part disappears, and the cellular vesicular character returns—till, at last, the pulmonary tissue resumes its natural dryness and colour, though still, for some time, remaining firmer, more elastic, and heavier than healthy lung. Resolution, however, seldom returns equally in all the inflamed parts—some spots remaining harder than others for a certain time.

Even when pulmonic inflammation has reached the third stage, (purulent inflammation) Laennec affirms that resolution is possible, the pus becoming absorbed, without any disorganization of the lung remaining, except a degree of density. It is slightly crepitous, and does not always sink in water; but, when incised, the surfaces are still of a dirty-yellow, or green colour, very different from that of sound lung. If resolution be far advanced, this colour is the only remaining mark of previous inflammation. Before Laennec began to use emetic tartar in pueumonia, he had frequent opportunities of examining the bodies of those who died of the disease; but latterly he asserts that he lost very few indeed, except where there was another and more dangerous concomitant disease, as of the heart.

DURATION OF PERIPNEUMONY. Our author has frequently seen the first degree of acute peripneumony continue for seven or eight days, affecting the whole of one lung and part of the other, terminating fatally before the occurrence of distinct hepatization. Such result was very common in the epidemic of 1803—4. This was the case where bleeding had been largely used, and where it had not been used at all. In other cases, however, especially where pneumonia attacks debilitated subjects, or supervenes on other severe maladies, the third stage, or purulent infiltration, will sometimes take place in 36, or even 24 hours.

"With the exceptions just stated, I think we may fix the duration of the different stages of peripneumony as follows: the obstruction, or first stage, usually lasts from twelve hours to three days, before passing to the state of complete hepatization; this lasts from one to three days before spots of purulent infiltration make their appearance; and the period of suppuration (from the time when the concrete purulent infiltration is distinctly perceptible, until this is completely softened to a viscid fluid) varies from two to six days. Bloodletting, derivatives, and resolvents or stimulants of the absorbent system, obviously retard the progress of the disease, and consequently prolong the period of the first two stages. Convalescence is rapid in proportion as the inflammation is of small extent and has been early checked."

It may be observed, that the mucous membrane of the bronchia is generally very red in the inflamed portions of lung, and sometimes thickened.

### II.—SYMPTOMATOLOGY.

We now come to the physical signs, as revealed by auscultation, percussion, &c. and, also, to the common symptoms of pulmonic inflammation. Before morbid anatomy had made its recent advances, pneumonia was considered a disease very easily cognizable; but this is very far from being the case, except where it is uncomplicated and has made much progress. In its commencement, and when complicated with other affections, it is latent or masked, because its symptoms are common to other diseases as well as to pneumonia.

1. Physical Signs. The crepitous rattle is the pathognomonic sign of the first stage; being perceptible from the very beginning of the inflammation. At this time, it seems hardly to possess the character of humidity. The respiratory sound is still heard distinctly, combined with the crepitous rattle; and percussion affords the natural resonance. The extent over which the ear or the stethoscope detects the rattle or wheeze, indicates the extent of the inflammation. This is frequently not greater than the diameter of the instrument. The further we remove the cylinder from the point affected, the râle becomes more obscure, and ceases altogether at two or three inches from the inflammation. In proportion as the obstruction increases, and verges towards hepatization, the rattle becomes moister and less uniform—the respiratory sound, which formerly accompanied it, dies away; and, at last, as hepatization takes place, the rattle itself ceases to be heard.* At this period, the sound, on percussion, does not differ materially from that of health, unless the obstruction is very extensive, and verging on the second stage, that of hepatization, in which case, it becomes somewhat more obscure. This is, also, the case in pretty extensive engorgements of the lower portions of right lung, where the sound is always obscure, on account of the presence of the liver.

Such, then, are the physical signs of peripneumony in the first stage—the "râle crepitant" being unquestionably the surest indication of its existence. It obtains from the very beginning, and is not present in any other disease, except œdema of the lungs and pulmonary apoplexy, which are easily distinguished by their proper signs. M. Laennec thinks M. Andral in error, when he observes that the crepitous rattle sometimes exists in simple acute bronchitis. Of all the stethoscopic signs, the crepitous rattle is perhaps the most useful, in a practical point of view, as pointing out, in the very earliest stage, one of the most severe and most common diseases, thereby enabling the physician to apply the remedy with much more chance of success than even a few hours later.

When the inflammation has reached the second stage, the crepitous rattle and also the respiratory sound disappear—and this disappearance is often the only indication we have of the occurrence of hepatization. Bronchophonism (the sound of the voice coming through the cylinder, something like pectoriloquism) exists in certain cases, particularly if the inflammation is seated near the roots of the lungs, or in the upper lobes, where the tubes are largest. If the mucous rattle exists at the same time in the bronchia, hepatization renders it much stronger and more distinct. Such are the physical signs of hepatization—namely, in two words, the absence of the crepitous rattle and the respiratory murmur, together with a dull sound on percussion over the affected part, unless the pneumonia be central, with a layer of sound lung between it and the ribs, when percussion will not much avail.

---

* The crepitous rattle is supposed to be produced in the air-cells themselves, by the intermixture of air and fluid; in the same way that the mucous rattle takes place in the bronchia.

SIGNS OF SUPPURATION. The infiltration of pus within the pulmonary tissue furnishes no new sign as long as the pus remains concrete; but when it begins to soften, we perceive in the bronchia a more or less distinct mucous rattle, occasioned either by the introduction of the pus into them, or by a more copious mucous secretion, sympathetic of the suppurative process going on in the pulmonary tissue.

SIGNS OF ABSCESS. When the pus is not absorbed or expectorated as it softens down, but collects in one spot, a very strong mucous or cavernous rattle is perceived over the site of the abscess. Bronchophonism is converted into pectoriloquism, and the respiration and cough become cavernous.

SIGNS OF RESOLUTION. When this takes place, before the establishment of hepatization, the crepitous rattle becomes daily less perceptible, while the natural sound of respiration becomes more distinct, and at last is alone heard; and when hepatization does take place, its resolution is invariably announced by the return of the crepitous rattle. M. Laennec has never seen this sign wanting in any case which he has daily examined. He denominates it the "renewed crepitous rattle." The same renewal announces the resolution of the third stage or purulent infiltration; but in this last case, it is usually preceded by the mucous wheeze or rattle, indicating the softening of a part of the pus.

It is not to be concealed that there are some cases, in which the physical signs of pneumonia are with difficulty recognized—namely, when the inflammation is seated in the centre of a lung, or complicated with another disease. Laennec thinks, however, that these difficulties have been exaggerated by Andral. We fear that there are many others besides M. Andral who have experienced these difficulties. But still they are of the negative kind, and are rare. The signs may be wanting where the disease is present; but the disease will not be absent when the signs are present.

"Of all the affections of the organs of respiration which may be combined with pneumonia, the suffocative catarrh is unquestionably that which most masks its characteristic signs. If the pneumonia is of very small extent, and supervenes to the catarrh, it is possible that it may be masked by the presence of the very loud mucous rattle which exists over the whole bronchia. It is this circumstance which renders the *pneumonia of the dying* so difficult to be recognised."

As far as regards this complication with catarrhus suffocativus, (which is a very rare disease,) the difficulty of diagnosis is of no practical importance.

### III.—SYMPTOMS OF DISORDERED PULMONARY FUNCTION.

We now come to a portion of symptomatology to which the anti-auscultator cannot object. The following extract from Dr. Forbes's translation of Laennec is most valuable.

"These usually are—an obtuse and deep-seated pain, dyspnœa, quick respiration, cough, and expectoration of a peculiar kind. To these symptoms, decubitus on the affected side is commonly added; but nothing is more variable than this. The other symptoms are more constant, though each of them may be wanting, and even, in particular cases, all of them may be so at the same time. Moreover, they may all co-exist in many other diseases as well as pneumonia; and each of them exhibits many varieties.

Thus the pain, which is commonly slight and extensively diffused, is sometimes confined to a point, even when there is no accompanying pleurisy. However, when it becomes very acute, it is commonly on account of the inflammation being extended to some part of the pleura. The dyspnœa is often hardly perceived by the patient, although the frequency of the respiration points it out to the physician; in some cases this is not more frequent than in health. When dyspnœa does exist, the inspection of the chest will not enable us to decide whether or not it depends on an organic affection of the lungs; as the dilatation of the chest and the elevation of the ribs are often equal on the sound and diseased side,—a remark which has also been made by M. Andral, (Op. Cit. p. 330.) The cough is commonly frequent and pretty strong; but sometimes it is so slight as to be denied by the patient and the attendants. The expectoration in a great many cases has an appearance quite characteristic, and which, in my opinion, may by itself enable us to recognize the disease; as I have never met with it in any other. These sputa, which I shall term *glutinous* or *pneumonic,* when received into a flat and open vessel, unite into so viscid and tenacious a mass, that we may turn it upside down, even when full, without the sputa being detached, although they may partially hang from the vessel's mouth. If we shake the vessel its contents vibrate like jelly, but less so. The colour of this expectoration is frequently some shade of red, particularly that of rust; or it is sea-green, tawny, orange, saffron, yellowish or a dull green. These various colours are frequently intermixed, in stripes, in the same spitting-pot; and are evidently owing to blood existing in a greater or less proportion, or more or less intimately combined with the expectorated matter. The shades of green appear to me to depend on the same cause, although they constitute the bilious sputa of Stoll and his disciples. Certain it is that I have frequently met with them in cases where there existed no bilious complication; although I must admit, at the same time, that I have sometimes seen them disappear, after bilious evacuations. The entire body of the expectoration has a semi-transparency like that of horn; and sometimes it is almost as transparent as white of egg very slightly coloured. Air-bubbles, of unequal size and sometimes very large, are contained in great number in the expectoration, and cannot escape on account of its great tenacity. If sputa of this kind existed constantly in peripneumony, we should require no other sign to indicate its presence. They commonly appear in the stage of obstruction, and retain their character until hepatization is well advanced; they then vary much from the characters above-described, as we shall see presently. It is to be remarked, however, that, even in the first stages they do not always present the strongly marked features we have just described. Frequently they are less viscid, little coloured, and nearly destitute of air-bubbles; and at other times we perceive only a few *glutinous* and slightly tawny sputa, amid a great mass of mucous or pituitous expectoration. Pretty frequently, the characteristic sputa are observed only at the very onset of the disease, and during a few hours; and sometimes they do not show themselves even at this period, or they are in such small quantity as hardly to admit of being collected. This appears particularly the case in old subjects, and in very rapid attacks, and also in the peripneumony of the dying."

During the period of hepatization, the expectoration is slight and variable in character, usually consisting of a small quantity of pituitous sputa, more or less viscid and vitriform—or of whitish or yellowish mucus. After the purulent infiltration occurs, the expectoration is more decidedly mucous, resembling that in the latter stage of catarrh—rarely purulent. Lerminier and Andral consider an expectoration of sputa, apparently consisting of a mixture of blackish blood and diffluent pituita, as characteristic of the period of suppuration.*

---

* In this place Dr. Forbes takes an opportunity of impressing on the mind of the practitioner the necessity of paying great attention to the expectoration. Many are in the habit of minutely observing little varieties in the pulse, tongue, stools, &c.

GENERAL SYMPTOMS. Pneumonia is, of course, attended with active fever from the beginning, except in very rare cases, and where the inflammation is very limited. This general fever accounts for the flushings of the face, and the various sanguineous and serous congestions which occasionally take place in the brain and intestinal canal. Determination to the head, with coma, especially in old people, is a very unfavourable symptom, as has been remarked since the days of Hippocrates. In such cases the patient generally dies before hepatization takes place, or the purulent infiltration commences. A furious delirium, Laennec observes, is a much less dangerous symptom than coma. We believe this is the case in all other diseases as well as in peripneumony. In furious delirium, we have something to work upon, but in coma it is the reverse. "Congestion of blood in the stomach is indicated by a very intense redness of the tongue," with but little pain at the epigastrium when pressed. Diarrhœa is not considered a bad symptom by Laennec, if it comes on towards the close of the disease, and is moderate. The fever in peripneumony is truly symptomatic—that is to say, it depends on the inflammation of the organ, and rises or falls with that inflammation. "It even frequently happens, that as soon as this latter (inflammation) is checked by the lancet or otherwise, the fever ceases entirely, although the perfect resolution of the pulmonary engorgement will not be accomplished in less than a fortnight, three weeks, or even a month." There are cases indeed where the fever continues after the local inflammation ceases, but then there is some other cause, and the fever is idiopathic.

During the acute stage the urine is of a very deep red colour—the blood from a vein quickly coagulates and exhibits a thick coat of fibrin, especially at the first bleedings.

M. Laennec avers, that acute peripneumony frequently terminates favourably by a kind of spontaneous crisis, where either the disease had been mistaken, or where the remedies had proved useless. The most common of these critical evacuations is a lateritious sediment in the urine; "and we should distrust any other, unless this also occurs at the same time." After this deposition, a sweat, and a moderate diarrhœa, are the most common forms of crisis. "A copious expectoration of mucus is also sometimes critical, but much less frequently than the practitioners of the last century believed, unless, indeed, it be in those cases which occur during the course of a catarrhal epidemic." Upon this passage we would remark, from long experience, that whether the expectoration be *critical* or not, it is decidedly salutary—so much so, that we very rarely find the disease give way without this evacuation, unless the depletion be of an extremely vigorous kind at the very commencement of the pneumonia. The following observations, from that talented physician, the late Dr. Parr, we can confirm from personal experience.

"If peripneumony proceeds (says he) favourably, the pulse becomes slower and softer—the yellow, tenacious, and often bloody sputum is mixed with points of a whiter matter—and the proportion of this more salutary expectoration gradually increases with the amendment of every symptom; for the cough is less violent and straining, the breathing freer, the skin more moist, and the tongue cleaner at the edges. If less favourable in its progress, the sputum becomes more dark and viscid,

---

while they disregard the qualities of the expectoration. A spitting-pot is an essential article in the bed-room of a pulmonic patient, and where auscultation is imperfect, the young practitioner will draw much useful information from daily inspection of the matters ejected from the lungs.

the pulse lower, indistinct, and often intermitting—a wandering low delirium comes on, with subsultus, and the patient dies, apparently suffocated, from the oppressed vessels no longer permitting the expansion of the lungs."

We think, upon the whole, that Laennec has directed his attention too much to the auscultic signs, and passed too slightly over a careful and hippocratic observance of the general and concomitant phenomena. At the same time, we freely admit that a comparison of his diagnostics in pneumonia, with those of our standard authors, leaves the latter completely in the shade. We now proceed to the etiology of peripneumony.

OCCASIONAL CAUSES. One of the most common of these is, unquestionably, the impression of COLD, either long-continued, or received when the body is moderately heated and covered with perspiration.

"This cause (says Laennec) is much less powerful when the cold immediately succeeds to an excessive heat, and is not prolonged for a considerable time. The Russian who rolls himself in the snow after coming out of the hot bath, or the bakers who go from their heated ovens, almost naked, into an atmosphere of the temperature below zero, are not liable to attacks of this disease; while the porters, whose occupation leads them to stand for a length of time at the corners of the streets, are frequently affected by it. In general pneumonia is a disease of winter and cold climates: it is rare in the equatorial regions. The poison of serpents, particularly that of the rattle-snake, frequently induces this disease, and the same result follows the injection of various medicamentous substances into the veins. It is probable that the epidemic peripneumony is often owing to an analogous cause, that is to say, to deleterious miasms which have entered the system by means of the cutaneous or pulmonary absorbents; since nothing is more common than to meet with cases of this disease to which we can assign no occasional cause. How many persons are seized with it in their very chambers, and in spite of the utmost care taken of their health! Most pathologists reckon fulness of blood, youth, manhood, and a strong constitution among the predisposing causes of pneumonia. It is no doubt true, that, in subjects possessing these conditions, the inflammation is more acute, the fever higher, and the disease more readily recognized and cured; but it is no less true that peripneumony is much more common and fatal in old persons: it is in such subjects, more particularly, that the disease runs rapidly into suppuration. Children are likewise very subject to it, and the more so the younger they are. In them the disease is frequently mistaken, because they swallow the expectoration; and death most commonly takes place in the state of engorgement, or after the supervention of only a lobular hepatization, that is to say, a hepatization occupying only some detached points. The facility with which they fall victims to this affection even in its onset, is explained by the greater necessity of respiration at this period of life."

It is wonderful that so accurate an observer as Laennec, and so talented a physician as his translator, should have taken no notice of the application of MOISTURE to the surface, as the most common of all the causes of acute and indeed of chronic pneumonia. It is true, they may say that wet only increases the degree of cold, on the principle of evaporation. But there is something more than this in the case. Damp sheets, exposure to rain on the outside of a coach, or in various naval and military duties, excite this inflammation in a very intense manner, and far beyond what the mere degree of cold can account for. When we contemplate the deleterious effects of the application of cold and moisture to the skin, we can hardly doubt that these agents act directly on the respiratory organ at the same time. If we enquire diligently into the history of cases of peripneumony, acute and chronic, we shall rarely fail to find that moisture has accompanied the application of cold. These observations are not without utility in a practical point of view. Such articles of cloathing as prevent the penetration of moisture to the surface of the body, are of great importance in the way of hygiene. On this account, wash-leather is superior to flannel; though both should be used by those who are disposed to pulmonic inflammation, and who, from their necessary avocations, are exposed to wet and cold. A foolish and unfounded prejudice has long been entertained against oil-silk, as an ingredient in the more external articles of dress. We can vouch for its utility as a *safe* defence from moisture.

GANGRENE OF THE LUNGS. We shall pass over this formidable disease, because it is very rare, and because "it can scarcely be ranged among the terminations of pulmonary inflammation." M. Laennec thinks that, in most cases, it approaches the nature of idiopathic gangrenes, "such as anthrax, malignant pustule, pestilential bubo, &c.—diseases in which the inflammation surrounding the gangrenous spot seems to be rather the effect than the cause of the sphacelus." We have given some cases of this disease, and we shall continue to do so; but we think it unnecessary, if not improper, to introduce the subject formally in the present eclectic review of peripneumony. The only remark we shall make is, that an expectoration of a very peculiar green colour, and exhaling an extremely fetid odour precisely similar to that of a sphacelated limb, seems to be the principal pathognomonic sign of this dangerous disease.

### IV.—CHRONIC PERIPNEUMONY.

It is curious that the ancients make no allusion to this disease, while the term is hardly to be met with in the schools of modern times, unless it be in discussing the subject of phthisis pulmonalis, with which the disciples of Broussais connect chronic inflammation of the lungs. This connexion is strongly opposed by Laennec, who considers the chronic form of the disease as extremely rare. This observant physician has noticed a state of lung in the vicinity of gangrene, and also of the seat of hæmoptysis, which indicated chronic inflammation. The same condition of parts was seen, but still more rarely, in the neighbourhood of large tuberculous excavations, and also in the small interspaces between numerous tubercles. In all these, however, it is much more common to find the marks of acute hepatization, which had occurred only a few hours before death. We may also, thinks Laennec, term those cases chronic, in which the peripneumony, originally acute, has been checked by proper treatment, but not completely resolved. The following note of Dr. Forbes we shall here introduce.

"The statements contained in this section will, no doubt, appear singular to many English readers; and I confess that if I felt justified in placing the dissections made in this country (including my own) on the same level, as to minuteness and accuracy, as those made by the French pathologists, I should feel disposed to question the truth of these statements. But as every candid person must admit that the hurried manner in which dead bodies are commonly examined in this country, renders mistakes extremely probable; and as we must likewise confess that our means of observation, and consequently our experience, fall vastly short of theirs, it is perhaps no great stretch of candour to be willing to receive the authority of such men as Laennec, Chomel, Andral and Louis, in preference not only to many of our recorded cases, but even to our own hurried observation. The correctness of our author's statement respecting the great infrequency of peripneumony in a chronic form, is supported by the concurring testimony of the most experienced pathologists of the present French school. Andral says that of one hundred and twelve cases observed by him, only one lasted more than thirty days; and that during the five years which he had been at *La Charité*, he had met with very few examples of hepatization or purulent infiltration, in cases of more than two months' standing. (Med. Clin. t. II. p. 365.) M. Chomel states (Dict. de Med. t. XVII. p. 252) that in the course of the last sixteen years, during which he has examined, on an average, two hundred bodies annually, he has only met with two well marked cases of this affection. Andral notices it as existing under two forms, the *grey* and *red induration*, and describes it briefly as being dry and hard, of a pale red or greyish. (Op. Cit. p. 310.) Chomel describes the lesion in the two cases met with by him, as consisting of a grey, dense induration, without granulations, much drier and harder than hepatization, and occupying a fourth or fifth part of one lung. The same condition of lung is, I think, described by Corvisart, in his Commentary on Avenbrugger, p. 287, and by Avenbrugger himself, p. 262: and appears to be that found by myself in the case of chronic pleurisy detailed in "Original Cases," p. 247. M. Andral likewise considers that black induration of the lungs, sometimes existing around tuberculous excavations, and which Laennec describes as a particular degeneration under the name of *melanosis*, as being frequently the result of chronic inflammation simply. (Op. Cit. III. p. 230.) In the small work above-mentioned I have entitled several observations 'chronic peripneumony;' and I certainly have been in the habit of considering many cases I met in practice as examples of this disease. I am willing to admit however that I have been sometimes mistaken, and, both in practice and in my dissections, have confounded different affections under this name.

"The truth seems to be, that inflammation of the pulmonary substance, strictly and essentially chronic, (like the chronic affection of the serous membranes,) is extremely rare; but that, as a sequel of the acute disease imperfectly resolved, or as complicating other organic lesions of the lungs, it is by no means uncommon. Our author himself admits that the acute disease, made chronic by treatment, may last two months; and Lorinser says that this period may be doubled. Both M. Andral and M. Chomel, however, are of opinion that chronic inflammation of the pulmonary substance is very common under another name and form. They consider the thin layer of grey substance which is found surrounding softened tubercles (and which Laennec regards as simply tuberculous) to be the product of chronic inflammation."

### V.—LATENT AND SYMPTOMATIC PERIPNEUMONY.

Our author has already shewn, that it is sometimes extremely difficult to detect acute peripneumony in the first days of its attack;—it is, however, in its complications with other diseases, that it becomes still more masked, and likely to elude the notice of the practitioner—especially of him who trusts entirely to external symptoms.

The combination of peripneumony with pleurisy, is now passed over, as the latter inflammation will form a short article for consideration in a future fasciculus or number of the Journal.

Peripneumony is sometimes conjoined with hæmoptysis, and still more frequently with œdema of the lungs. The sero-sanguineous congestion which takes place in almost all dying persons, is very often converted into peripneumony, if the dying efforts are long protracted. On examination, different points of lung are found distinctly hepatised, especially during the prevalence of an inflammatory constitution. This "pneumonia of the dying" is commonly accompanied by a strong and suffocating tracheal rattle—but the presence of this rattle does not always indicate the existence of the disease. Pneumonia is occasionally combined with the different varieties of catarrh—" but more rarely, perhaps, than with any other disease of the chest." Laennec avers that it is by no means common to find the acute catarrh terminate in peripneumony. But the suffocative catarrh, especially in young people and adults, is often complicated with pneumonia.

" Phthisical subjects are liable to attacks of pneumonia, usually of small extent, and the symptoms of which are therefore very readily confounded with those of the primary disease. On this account, if for no other reason, it is important to explore, from time to time, the chest of consumptive patients, more particularly when there is any increase of fever, or any sudden decrease of strength.*—Several diseases which may be considered of a general kind, have a singular tendency to the peripneumonic complication, or to excite this affection sympathetically. It is thus found occasionally to supervene to an attack of gout or rheumatism. If the pains of the limbs cease on its attack, it is usually recognised, or at least suspected, from obvious symptoms, but if the pains continue, the pulmonary affection remains latent, or is only discovered by means of an attentive exploration. The eruptive fevers are sometimes combined with pneumonia. Measles, in particular, frequently presents this union, at the period of the disappearance of the eruption. In this case the pneumonic affection is pretty frequently manifest; but when it supervenes in the course of confluent small-pox, or severe erysipelas, it is almost always latent. The same is true of the pneumonia which arises in the course of violent continued fevers. Nothing is more common than this last named complication, especially in winter and during the prevalence of peripneumony; and in these cases, its invasion is seldom indicated by any unusual dyspnœa or expectoration, or, in short, by any of the ordinary symptoms of inflammation of the lungs. It is true that it only occurs towards the fatal termination of the disease ; but probably it is also very often the cause of this. In the young and robust, the invasion of the pneumonia may sometimes be suspected from a marked increase of fever taking place. But in old persons, and in subjects weakened by the long continuance of high fever and low diet, it comes on all at once, attended by a sudden prostration of strength and loss of consciousness. The skin becomes harsh, the excretions fetid, the teeth and tongue covered with a fuliginous coating, and coma or the tracheal rattle announce the approach of death. These latter symptoms frequently indicate the supervention of pneumonia in subjects worn out by severe chronic disease, especially cancer. We ought to range among sympathetic peripneumonies that which constitutes the predominant symptom in the pernicious fevers denominated peripneumonic. The morbid anatomy of this affection is yet very imperfectly known, from the circumstance of its proving rarely fatal! as we fortunately possess in cinchona, when administered in time, a certain means of cure. We have some facts, however, which prove that traces of pneumonia have been found in subjects dead of this disease; I have myself, in two accessions of this fever, witnessed the presence of the glutinous sputa, and a very intense crepitous rattle."†

---

* " Andral describes the intercurrent peripneumony of phthisical subjects as being very common, and as often occasioning death, from being overlooked. In the acute form it is remarkable for its frequent recurrence in the same subject, it being by no means uncommon to find the same patient affected with it twelve or fifteen times. (Cl. M. III. 225.) Louis, (Rech. p. 241,) while he admits the occurrence of this complication with phthisis in the early stages, (when it is most commonly cured,) notices it chiefly as supervening towards the very last days of the disease. At this time it is very frequent, yet, he says, not more so than in other persons dying of chronic affections, (see the preceding note ;) so that he conceives himself justified in stating that phthisis in its latter stages, has no particular influence in exciting peripneumony."—*Transl.*

† " A very important variety of pulmonic inflammation, important no less from the causes which give occasion to it than from its peculiar characters, has been lately introduced to the notice of the profession by some of our distinguished surgical

### VI.—TREATMENT OF PERIPNEUMONY.

In pneumonia, as in other inflammations, the indications of treatment would appear sufficiently obvious; and yet the most opposite measures have, in their turn, been held up to exclusive recommendation by those who have adopted particular theories. Laennec contents himself with an exposition of the results of his own experience—and, in following him, we shall have an opportunity of glancing at the experience of others.

BLOOD-LETTING. From the days of Hippocrates, this measure has been considered as peculiarly appropriate in pneumonia, with the exception of a few theorists, who have questioned its propriety. But there is considerable difference as to the extent to which this remedy should be carried—the best period for its employment—and the part from which blood should be abstracted. The majority of the ancients bled very early in the disease, and allowed the blood to flow till syncope took place. This practice was sometimes followed by Galen—it was much adopted about a century ago—and is still very common in England, "where practitioners bleed, at the commencement of pneumonia, to 24, 30, or 36 ounces." "This practice," says M. Laennec, "is not to be found fault with, since it is certain that a copious bleeding in the beginning of the disease, reduces the inflammatory orgasm much more speedily than repeated smaller venesections will do at a later period, and, moreover, leaves less chance of a renewal of the inflammation." It is wonderful, as Dr. Forbes remarks, that, after this statement, Laennec should not recommend the English practice, in preference to that generally followed on the Continent. We may observe, however, that the Parisian physicians—we may instance M. Lerminier, at La Charité—are rapidly adopting the system of very active venesection in the first days of acute inflammation. The following observations of Dr. Forbes are deserving of attention.

---

pathologists: I allude to that which supervenes to wounds and the larger operations, and which is, I fear, too often latent. See Guthrie's Treatise on Gun-shot wounds, (first published in 1815,) 2nd Ed. p. 284; and C. Bell's Surgical Observations, Part III. p. 241, Lond. 1817. From the statements made by these authors it appears that pneumonia is a very frequent cause of death in the cases in question; and that it comes on in the most insidious manner, scarcely giving warning of its presence, certainly not of its violence, until too late for beneficial treatment. In these cases I would strongly recommend the stethoscope to the surgical practitioner, as a sure, and almost exclusive, means of acquiring an exact knowledge of the progress of the disease. From the account given of it by Mr. Guthrie and Mr. Bell, it appears evident to me that had this instrument been applied on the first appearance of the dyspnœa, the crepitous rattle would have immediately pointed out the presence of the inflammation, of which the general symptoms gave little or no indication, and might have thereby been the means of checking its fatal progress by suggesting the proper remedies. At the same time that I state this, I am not ignorant that cases occur (though very rarely) so completely latent as not only to be unaccompanied by dyspnœa, cough, or expectoration, but even to yield no results from percussion or auscultation, (See Andral, t. II. p. 369.) The reason of the lungs becoming affected in the class of cases just noticed, is an interesting subject of inquiry, but one on which I cannot here enter."

"To the readers of this work it is unnecessary to say, that the quantity of blood mentioned in the text may be detracted twice, or even thrice, within the period of twenty-four hours, in the beginning of the disease, not only with safety, but unquestionable benefit—due consideration being had to the severity of the attack, the constitution of the patient, and the character of the prevailing epidemic. It is only in the more advanced stages of the disease, that greater caution is necessary in the detraction of blood; and it is the prosecution of the same vigorous treatment at this later period, too common in this country, that is justly obnoxious to the criticism of foreign practitioners. In such circumstances, there can be no doubt that the small bleedings and copious leechings used abroad are vastly preferable; or even the expectant system, with its starvation and its innoxious ptisans. The system of medical practice in this country is, perhaps, too generally chargeable with the imputation of over activity; the *medicina perturbatrix* is too exlusively cultivated, especially by the younger members of the profession. Poor Nature, with her *vis medicatrix*, is so scorned and outraged, in what, after all, is truly her own dominion, that it is no wonder if the acts of such radical reformers of her plans are sometimes turned to their own confusion. I believe, however, that the unlimited intercourse now happily existing among the nations of Europe, is gradually improving the medical practice of each individual country. This is obvious in respect to bloodletting in pneumonia. M. Andral, in his late work, says that the first bleeding should be from sixteen to twenty ounces, and that the operation may be repeated twice, or even thrice, within the first twenty-four hours (Op. Cit. tom. ii. p. 379). M. Chomel, also, in his article on pneumonia, in the *Dict. de Med.* (tom. XVII. p. 243), says that the first bleedings should be from twelve to sixteen ounces, and that one may be repeated a few hours after another, to the third time on the same day. For some excellent remarks on the propriety of instituting one very copious bleeding in the early stage of pneumonia, I refer the reader to a paper by Dr. Robertson in the Edin. Journ. vol. x. p. 192. The aphorism by Dr. Gregory there quoted—'the danger of a large bleeding is less than the disease'—is excellent; and it were well if it were more frequently in the recollection of practitioners, in the beginning of inflammatory diseases. Without at all sanctioning the practice therein detailed, I would also refer the reader to a singular document in the same journal (vol. XIII. p. 165), for proofs of the astonishing extent to which bloodletting may be carried with safety at least, if not with benefit. The writer, Mr. Comrie, states that his practice (the disease was the ardent fever of the West Indies, the patients seamen) was, to take away fifty, sixty, or seventy ounces of blood at the first bleeding; and that his patients sometimes lost one hundred ounces within the first twelve hours, and upwards of two hundred and fifty ounces in the course of three or four days! I once knew a man bled to eighty-four ounces at one bleeding, in an attack of fever, without suffering syncope, or any ill effect, except great disorder of the circulation for some hours afterwards.—*Translator.*"

It was in our fleets and armies, during the late war, that peripneumony was seen on a large scale, and where that decisive practice was adopted, which afforded many salutary lessons to our more timid practitioners in private life. We shall not apologize for the following extract from a report, published by the Editor of this Journal, in February, 1814, from the fleet blocked up in the Scheldt, during the dreadful winter of 1813-14. It will shew that the cautions of Dr. Forbes, as to large bleedings at an advanced period of pneumonia, were not unattended to by his naval brethren.

"There is perhaps no acute disease, where Nature proves a worse physician, than in pneumonia. In this, she seems to attempt a relief of the local plethora and inflammation by *expectoration*, in the same way as she attempts the cure of dysentery by *catharsis*. She is not very successful in either; but less so in the former than in the latter case. This *natural* method (if I am allowed the expression) of cure, has led many people to trust too much to expectoration, by directing a great deal of their attention to bringing on this salutary process. Those who have seen pneumonia on a large scale, know, that very frequently the most violent cases of it are cured, as it were, 'by the first intention,' where the inflammation has been quickly subdued, and the vessels of the lungs relieved, without the necessity for that effusion of fluid into the ramifications of the bronchia, constituting expectoration.

"Venesection, from a large orifice, *continued till relief of the pain and dyspnœa, or deliquium animi, as early as possible after the accession of the disease,* is the first step; and the next is, to empty the bowels effectually, by a purgative that will occasion a considerable effusion into the intestinal canal, from the whole of its mucous surface. The third, is to relax the surface by saline draughts and antimonials. The two first are to be repeated boldly and quickly, *in the early stages particularly;* and while dyspnœa and pain are urgent. *But after two or three days, and when any expectoration, however scanty, appears, we must immediately relax in our vigorous measures; for we have failed in subduing the inflammation by the first intention, and by pushing the evacuating plan too far, we counteract Nature in the process which she has now employed for completing the cure.* I am convinced I have seen many cut off by bleeding profusely after the first days of the disease; thus checking expectoration, and occasioning such an effusion into the cells of the lungs as has speedily suffocated the patient. At the critical period above-mentioned, *blisters and local bleedings* are of the highest service; and nothing is more common than to see young practitioners apply the former, on the first or second day of pneumonia; when, after two, three, or four copious venesections, and other evacuations, they would have come in as important auxiliaries. After a mild and copious expectoration had commenced, I have seen a single purgative, too rough in its operation, check, at once, this salutary process, and bring the patient to the brink of the grave. Another error into which young men are often led, is the employment of stimulating expectorants, such as gum ammoniac, squills, &c. too early. Indeed, they oftener do harm than good; and, except in advanced stages, when the inflammatory diathesis is pretty well gone off, and a troublesome dry cough, with scanty expectoration, remains, they are rarely admissible."—*New Med. and Phys. Journal, February,* 1814.

But to return to M. Laennec. The ancients, says he, considered bleeding as a questionable remedy, after the first days of the disease, fearing thereby to check the expectoration; "and the best practitioners of the two last centuries forbad this operation after the fifth day, if the discharge was mucous and abundant. Apprehensions of this kind are not, perhaps, unreasonable, if the loss of blood is carried to syncope, but we know from experience that, in a lesser degree, though still pretty copious, blood-letting may be had recourse to with much advantage, in a very advanced period of pneumonia, even when this has reached the suppurative stage, and is attended with a great expectoration." Notwithstanding that many eminent authorities, and, among others, Andral, support this doctrine, we have no hesitation in impugning it. We do not consider the French as good judges on this point; for, till very recently, they did not bleed in a proper manner, even in the very first stage of pneumonia. In the second place, why should blood be drawn at a late period of the inflammation, and when there is a copious expectoration, unless the breathing is much oppressed, or there is considerable pain in some part of the chest? Yet we do not hesitate to assert, that when the "expectoration is copious," the breathing is almost invariably relieved; and, in such circumstances, local pain is generally the result of some local inflammation of the pleura, which may be removed by leeching, cupping, or even by blisters, without the risk attendant on venesection. We repeat it, then, that after copious, and more especially muco-purulent, expectoration has commenced, nothing but serious oppression of the breathing should lead the practitioner to employ general blood-letting—and this oppression of the breathing not being urgent, pain at such periods should be remedied by local bleeding, or rather by blisters. The following extract from one of Dr. Forbes's notes, will corroborate this opinion.

"To the testimony of facts we can oppose no equivalent objection; although, considering the very limited powers of art in removing great alterations of structure, it might be reasonably conceived, *à priori*, that a hepatized lung was not likely to be much under the influence of venesection. This much, at least, I am justified in stating from my own experience, that the vastly inferior power of bleeding in the second and third stage of pneumonia, ought to make us depend principally upon what we can effect in the first stage. And as guiding our practice in this most important particular, I consider the stethoscope as of the utmost consequence; for, without it, who shall say positively that the disease is in its first or its second stage? On this point of practice, the opinion of Lorinser is strongly against bleeding in the latter stages. He says that, after hepatization has taken place, bleeding, by weakening the powers of the system, impedes or altogether prevents the absorption of the effused lymph; and that while one or two venesections in the first stage, often suffice to produce complete resolution, six or even ten, in the latter stage, will not only have no good effect, but will decidedly hasten the fatal event. He adds that he has repeatedly proved the truth of this doctrine in the epidemic pneumonia of cattle, (*Lungenseuche der Rinder*) in which he invariably found bloodletting if not injurious at least useless, after the disease had reached the stage of hepatization."

M. Laennec observes, that the present practice all over Europe is—"in the beginning of the disease, to bleed to the extent of from eight to sixteen ounces, and to repeat the operation daily, and sometimes even twice a day, if the inflammatory symptoms do not give way, or if, after being subdued for a few hours, they return with fresh violence. After the first five or six days, the bleedings are repeated after longer intervals, and soon cease altogether, except in cases where they are strongly indicated by the renewed strength of the pulse, oppression, and fever."

If this be the practice on the Continent, we maintain that it is wrong. It certainly is not the practice among the more intelligent practitioners of this country—and we have reason to know, that a more active early treatment obtains in many parts of Italy and Germany. From a pretty long and extensive experience in this class of inflammations, we can conscientiously recommend the medical attendant, when called early to acute pneumonia, to abstract blood, in the recumbent position, till a full inspiration can be taken without inconvenience, or exciting cough—or, till syncope take place. When either of these effects is produced, he may bind up the arm, and persevere with the other antiphlogistic measures, presently to be detailed, till the pulse rises, the respiration becomes impeded, and fever and pain increase. Then the vein is to be re-opened, whether at the end of two or of twelve hours, and the same objects are again to be obtained, even at the expense of syncope. This mode of procedure should be carried on during the first days of the inflammation, if the inflammation continues for days, especially in subjects whose constitutions are not impaired by age or by other maladies. By this plan, we have repeatedly arrested, and seen arrested, the most intense inflammations of the lungs, with little, and sometimes no succeeding expectoration. In fact, the more active the treatment in the early stage, the less necessity will there be for expectoration, which is the process that Nature employs to remove the effects of the inflammation—namely, the engorgement of the vessels of the lungs. When this process has once commenced, we certainly should be cautious in the employment of the lancet.

So much for venesection. It is not a little remarkable that Laennec does not even mention local depletion. Now, one great advantage of auscultation and percussion is to indicate the locality of the inflamed part, in order that local depletion and counter-irritation may be employed as near that part as possible. Blood, therefore, should be taken from the chest by leeching or cupping, simultaneously with, or soon after general bleeding, by which means, the repetitions of the latter will often be considerably abridged. After one local, and one or two general bleedings, a blister may be applied, but not sooner. Much mischief is often done by the early application of blisters, before the vascular system is considerably emptied.

M. Laennec adverts to the pneumonia which is complicated with typhoid and other fevers, and where depletion cannot, of course, be carried to the same extent as in pure pulmonic inflammation. The remains of our army after the battle of Corunna, and the French prisoners in our pontons, during the late war, presented a wide field for observation in this dreadful complication of idiopathic fever with pneumonia, and if Mr. Lawrence had been on this field, he would have been taught, by dire experience, that neither fever nor inflammation can be "put out" (an expression very aptly used by Mr. Brodie, and very injudiciously ridiculed by Mr. Lawrence) by any system of depletion, however early or however vigorously employed. It was tried by many and by untimid practitioners, at the period alluded to—especially among the prisoners of war; but, for one case where the fever and inflammation were "put out" by such measures, there were ten where the patient's life was "put out" with the disease! These facts must be borne in mind, when we have to treat the pneumonia that arises during idiopathic fever. We repeat it, that it cannot be "put out," by vigorous general depletion. We must trust almost entirely to local depletion and counter-irritation in such cases.

Although the pulse is less fallacious in peripneumony than in most other internal inflammations; yet it is sometimes deceptive, and Laennec has given us a very useful precept on this point. Where there is doubt about the indication afforded by the pulse, the ear or the stethoscope should be applied to the region of the heart. Whenever the pulsations of the heart are proportionally much stronger than those of the arteries, then, he avers, we may bleed without fear. If, on the other hand, the action of the heart as well as that of the arteries be weak, we should be very cautious how we abstract blood from the general system. This, we hope, will prove a valuable precept in other diseases than peripneumony.

BLISTERS. Laennec seems to have little or no confidence in these derivative auxiliaries. He limits their application to those cases in which resolution proceeds too slowly, after the first stage is over—and to the chronic form of the disease. He thinks that a blister applied to the chest is injurious, by impeding the action of the respiratory muscles. We cannot but think this objection futile, though we acknowledge the bad effects of blisters too early applied.

EXPECTORANTS. Much has been written on this class of medicines, but little is known on the subject. The alkalies have been greatly lauded by Mascagni and others, as having a powerful tendency to promote expectoration. Dr. Farnese, a pupil of Mascagni, was in the habit of giving from a drachm to an ounce of carbonate of potash daily, dissolved in half a pint of water. We doubt whether such doses might not materially injure the stomach and bowels, by too rapidly dissolving the mucus on their internal surface, and exposing their nerves too freely to the action of food, physic, and certain stimulating secretions, as the bile. For our own parts, we have no confidence in any expectorant but antimony, aided and preceded by blood-letting.

PURGATIVES. Laennec advises to keep the bowels open in pneumonia, "especially on the approach of convalescence," by glysters and gentle laxatives. Dr. Forbes accords in this rule, and deprecates purgation. Our own experience leads us to the same conclusions in respect to purgation as to general blood-letting. In the early stage, when inflammation runs high, the bowels may be freely acted on; but in proportion as expectoration comes on, we must be cautious of purging, since hypercatharsis very readily checks the expectoration, and places the patient's life in imminent jeopardy. It is in such cases the carbonate of ammonia has considerable power in renewing expectoration.

TARTAR EMETIC. This remedy, though long in use, both in England and other countries, as an expectorant, in small, but nauseating doses, has lately acquired great celebrity, in pneumonia especially, when exhibited in comparatively large doses. Before making any observations on the mode of administration which we have found most advantageous, we shall lay before our readers the plan of Laennec, who seems to think that this medicine may almost supersede the lancet in pneumonia.

"As soon as I recognize the existence of the pneumonia, if the patient is in a state to bear venesection, I direct from eight to sixteen ounces of blood to be taken from the arm. I very rarely repeat the bleeding, except in the case of patients affected with disease of the heart, or threatened with apoplexy, or some other internal congestion. More than once I have even effected very rapid cures of intense peripneumonies without bleeding at all; but, in common, I do not think it right to deprive myself of a means so powerful as venesection, except in cachectic or debilitated subjects. In this respect M. Rasori does the same. I regard bloodletting as a means of allaying, for a time, the violence of the inflammatory action, and giving time for the emetic tartar to act. Immediately after bleeding I give one grain of the tartar emetic, dissolved in two ounces and a half of cold weak infusion of orange-leaf, sweetened with half an ounce of syrup of marsh-mallows or orange-flowers; and this I repeat every second hour for six times; after which I leave the patient quiet for seven or eight hours, if the symptoms are not urgent, or if he experiences any inclination to sleep. But if the pneumonia has already made progress, or if the oppression is great, or the head affected, or if both lungs or one whole lung is attacked, I continue the medicine uninterruptedly, in the same dose and after the same intervals, until there is an amendment, not only in the symptoms, but indicated also by the stethoscopic signs. Sometimes even, particularly when most of the above-mentioned unfavourable symptoms are combined, I increase the dose of the tartar emetic to a grain and a half, two grains, or even two grains and a half, without increasing the quantity of the vehicle. Many patients bear the medicine without being either vomited or purged. Others, and indeed the greater number, vomit twice or thrice, and have five or six stools, the first day; on the following days they have only slight evacuations, and often indeed have none at all. When once *tolerance* of the medicine (to use the expression of Rasori) is established, it even very frequently happens that the patients are so much constipated as to require clysters to open the body. When the evacuations are continued to the second day, or when there is reason to fear on the first that the medicine will be borne with difficulty, I add to the six doses, to be taken in twenty-four hours, one or two ounces of the syrup of poppies. This combination is in opposition to the theoretical notions of Rasori and Tommasini, but has been proved to me by experience to be very useful. In general, the effect of tartar emetic is never more rapid or more efficient than when it gives rise to no evacuation; sometimes, however, its salutary operation is accompanied by a general perspiration. Although copious purging and frequent vomiting are by no means desirable, on account of the debility and the hurtful irritation of the intestinal canal which they may occasion, I have obtained remarkable cures in cases in which such evacuations had been very copious. I have met with very few cases of pneumonia where the patient could not bear the emetic tartar; and the few I have met with occurred in my earliest trials; insomuch that this result now appears to me to be attributable rather to the inexperience and want of confidence of the physician, than to the practice. I now frequently find that a patient who bears only moderately six grains with the syrup of poppies, will bear nine perfectly well on the following day. At the end of twenty-four or forty-eight hours at most, frequently even after two or three hours, we perceive a marked improvement in all the symptoms. And sometimes even, we find patients, who seemed doomed to certain death, out of all danger after the lapse of a few hours only, without having ever experienced any crisis, any evacuation, or, indeed, any other obvious change but the rapid and progressive amelioration of all the symptoms."

Our readers will perceive that one common effect of this antimonial treatment is vomiting and purging at the beginning; and as the latter evacuation is not considered injurious by Laennec, it confirms the observations we made on early purgation, and nullifies the timidity evinced by himself, and also by his translator, in the preceding pages. Having no experience in this mode of administering emetic tartar, we leave it for the consideration of our readers. If it maintains the character drawn of it by Laennec, it must prove a valuable auxiliary, or even succedaneum to venesection, where it is desirable to husband the vital fluid.

The manner in which we have employed the remedy is as follows:—Immediately after venesection, the bowels are to be opened by a solution of Epsom salts, senna, and tartarized antimony; and, this done, the antimonial wine, in doses of from 30 to 60 minims, with fifteen drops of the vinum colchici, and five drops of tincture of digitalis, is to be exhibited in a common saline draught every four or six hours. If nausea be produced, the doses of the three ingredients are lowered—if no effect seems to result, they are increased. We have found such decided good from this mode of treatment, that we have not, as yet, ventured to change it for the Rasorian method. It must ever be remembered, however, that irritation, or even inflammation of the gastro-intestinal mucous membrane is frequently complicated with pneumonia. This is to be ascertained by the tongue, by the state of the secretions, and by the increased sensibility of the stomach and bowels to drink and medicine. When such complication exists, it renders the treatment more difficult. The antimonial medicines must be abandoned, and leeches should be applied to the epigastrium. We must trust then almost entirely to general and local blood-letting. It is curious that Laennec, a French physician, does not regard this complication as any bar to the administration of large doses of emetic tartar. His decided antipathy to the doctrine and practice of his countryman and rival, M. Broussais, appears to have led our worthy author into a dangerous kind of opposition on this point.

Before quitting the subject of treatment, it is proper to notice the plan first introduced by Dr. Hamilton, of Lynn Regis—namely, the administration of calomel and opium till the mouth is affected. His practice was, after bleeding and opening the bowels, to give from one to five grains of calomel, combined with from a quarter to a grain of opium, every six, eight, or twelve hours, according to the urgency of the symptoms, the patient being ordered to drink plentifully of diluent fluids in the mean time. The success attendant on this plan, "was such as to fill him with astonishment." The writings of Armstrong, and many others, have tended to confirm the utility of Dr. Hamilton's practice, with some modifications. When we consider the great influence of calomel and opium in resolving inflammation in the liver, the eye, the heart, and the serous and mucous membranes, we see no reason to doubt its power over inflammation of the parenchymatous structure of the lungs. The plan, therefore, should not be suffered to go into oblivion from prejudice. From the long established efficacy of this combination in dysentery, where there is unequivocal irritation, as well as inflammation, of the mucous membrane of the bowels, we have every reason to believe that it would be an important remedy in the complication of peripneumony with gastro-enteritis—a disease which is equally dangerous and difficult to manage by the common modes of treatment.

There is a state, near the conclusion of peripneumony, which embarrasses the young practitioner more than other periods of the complaint. It is that, in which the expectoration is going on favourably, and the patient is apparently in progress to recovery, but where, from sudden cause, the expectoration suddenly stops, and suffocation is menaced. The practitioner is undecided whether to give stimulants or to abstract blood. In such a state the loss of a few ounces of blood generally seals the patient's fate—and a few doses of carbonate of ammonia, in almond mixture, often restores the expectoration, when life is fast ebbing, and where nothing but this restoration of the process of expuition can save the patient. We have never found any medicine equal to the carbonate of ammonia in such cases; and we have seen many lives saved by its judicious administration. From five to fifteen grains should be given every hour or two till the expectoration recommences, when the dose should be lessened, and the intervals lengthened.

We now bring this article to a close. By means of small type, large page, and patient concentration, we have brought together a vast mass of important information on the subject of peripneumony—the laborious production of various practitioners in different and distant countries. This may serve as a specimen of the advantages which the public derives from the press—and especially from the periodical press. For little more than *four-pence sterling*, our readers have got the *substance* of some octavo volumes! We hope to make the remainder of our pages equally pregnant with useful practical matter, as those occupied with our first article, which is necessarily long, as the leading article of this quarter's number. The Review Department in the other Fasciculi will be in opener type, like that in the first page.

